# Supplementary material for: Quasi 2D electronic states with high spin-polarization in centrosymmetric MoS2 bulk crystals
Source: Sci Rep. 2016 Jun 1;6:26197. doi: 10.1038/srep26197 (PMC4887890; doi:10.1038/srep26197)
Supplement: Supplementary Information [file srep26197-s1.pdf]

Supplementary Information

Quasi 2D electronic states with high spin-polarization in centrosymmetric MoS_2 bulk crystals

Mathias Gehlmann[1], Gustav Bihlmayer[2], Irene Aguilera[2], Ewa Mlynczak[1,3], Markus Eschbach[1], Sven Döring[1], Pika Gospodaric[1], Stefan Cramm[1], Beata Kardynal[4], Lukasz Plucinski[1,*], Stefan Blügel[2], and Claus M. Schneider[1]

[1]PGI-6, Forschungszentrum Jülich GmbH, D-52425 Jülich, Germany
[2]PGI-1/IAS-1, Forschungszentrum Jülich GmbH and JARA, D-52425 Jülich, Germany
[3]Faculty of Physics and Applied Computer Science, AGH University of Science and Technology, al. Mickiewicza 30, 30-059 Kraków, Poland
[4]PGI-9, Forschungszentrum Jülich GmbH, D-52425 Jülich, Germany
[*]l.plucinski@fz-juelich.de

Figure 1 shows the comparison of ARPES spectrum along $\overline{\Gamma \mathrm{K}}$ direction to the corresponding calculation of bulk projected band structure for 2H-MoS_2 and 3R-MoS_2. The broadening of the valence band around the $\overline{\Gamma}$ point is a good indication for the delocalization of these states in the out-of-plane (ΓA) direction. The high dispersion is a result of the 3D bulk-like character of these states in contrast to the quasi 2D valence band around the $\overline{\mathrm{K}}$ point, which is discussed in the main article.

In figure 2 we analyze the contribution by the band structure of 2H-MoS_2 and 3R-MoS_2 quantitatively. Fig. 2 a)-c) show the ARPES spectra and DFT calculations that we discuss in the main article including markers that indicate the position of the energy distribution curves (EDCs), which are shown in Fig. 2 d)-f). The figures also show a least squares fit with the bulk projected band structure of the 2H and 3R-phase. Our DFT (film) calculations show no evidence for surface states in these energy- and momentum-regions. The fitted curve in Fig. 2 d) and f) are in good agreement with the data and show only 6.5% (d) and 0% (f) contribution of 3R. The fit in Fig. 2 e) to the data does not properly reproduce the data. Nevertheless, the agreement of the band structure of 2H-MoS_2 here is also by far better. The only point in the ARPES spectrum that seems to agree better with the calculations for the bulk projectet band structure of 3R-MoS_2 is highlighted with an arrow. However, our film calculations (main article) show a surface state in exactly this region.

In addition to the ARPES data we analyze the reflectivity spectrum in Fig. 3 by fitting a linear combination of reference spectra from Ref.[1] to our data. One can see that the sligtly different shape of the peak in our data at 2 eV compared to the 2H reference spectrum is compensated by a 8.7% portion of the 3R phase in the least squares fit. This, however, might as well be an artifact of the experimental setup since the shape of this peak is known to be asymmetric even for pure 2H-MoS_2.[2]

In summary we find no clear indication of the existence of a different phase in our sample and with the methods that we employed it is safe to assume 10% of 3R-MoS_2 as an upper limit. Figure 4 shows a calculation that estimates the effect of a mixed crystal phase on the spin-polarization that is observed in photoemission. As we discussed in the main article the observed polarization in 2H-MoS_2 is obscured by its alternating sign from one layer to the next. Consequently the initial spin-polarization that one can relate to the experimental observation depends on the probing depth, which we cannot reliably determine. Therefore we plotted the initial spin-polarization of the bands in 2H-MoS_2 that we can conclude from observing 65% in photoemission for several probing depths. In the main article we estimated 4 Å inelastic mean free path (IMFP) according to the minimum of the universal curve for photoemission. The blue curve Fig. 4 shows that for an IMFP of 4 Å, observing 65% spin-polarization corresponds to a fully spin-polarized initial state in each layer, which is in perfect agreement with our DFT calculations. Even for a possible contamination of our sample by up to 10% 3R-MoS_2 we still can conclude that the 2H-layers are at least 95% spin-polarized.

The plot also shows that assuming the minimum probing depth can only underestimate the initial polarization since a larger IMFP further conceals it. A further argument for the low IMFP is that the curve for 5 Å exceeds 100% . This is due to the fact that at this probing depth even with fully polarized monolayers the contribution of the opposing layers is to strong to

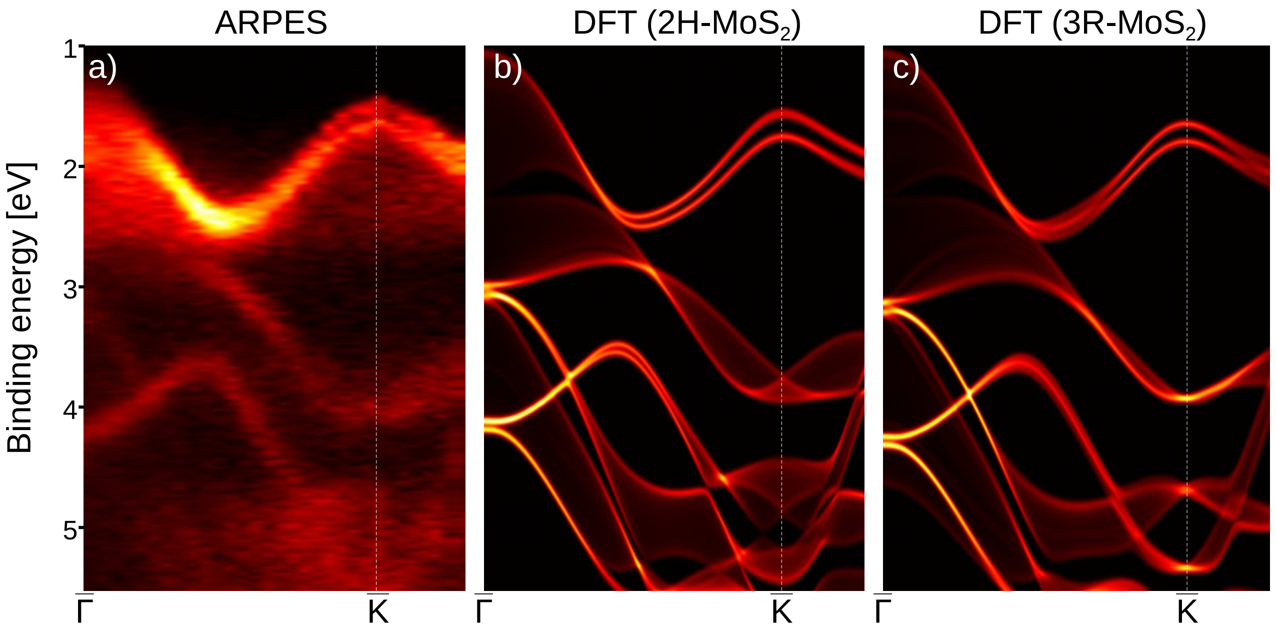

Figure 1. a) ARPES spectrum along $\overline{\Gamma K}$ direction measured with hν = 47 eV at BL5, DELTA and calculation of bulk projected band structure for 2H-MoS_2 in b) and 3R-MoS_2 in c). In the calculated maps bright areas indicate sharp bands with little out-of-plane dispersion, darker bands a broader projection. In b) and c) the Fermi energy is shifted for comparison with a).

observe 65% polarization as we did. In this case 23% of the 3R-MoS_2 could explain our observation, which is unlikely as we have shown.

One can see in figure 4 that for an IMFP of 4 Å (blue curve) our observation of 65% spin-polarization perfectly matches our DFT calculations, which show fully polarized layers in a pure 2H-MoS_2 crystal. If, however, the sample was in fact partially composed of 3R-MoS_2, whose spin-polarization does not alternate throughout the layers and is therefore independent of the probing depth, then already a lower degree of polarization in 2H-MoS_2 could explain our results. At a higher probing depth (yellow and red curve) the polarization of the 2H-phase is more obscured by the contribution of deeper layers. Therefore, in this case an even higher spin-polarization of the 2H-MoS_2 monolayers has to be assumed to explain our results.

References

1. Suzuki, R. *et al.* Valley-dependent spin polarization in bulk MoS_2 with broken inversion symmetry. *Nat. Nano* **9**, 611 (2014).
2. Beal, A. R. & Hughes, H. P. Kramers-Kronig analysis of the reflectivity spectra of 2H-MoS_2,, 2H-$MoSe_2$, and 2H-$MoTe_2$. *J. Phys. C.* **12**, 881 (1979).

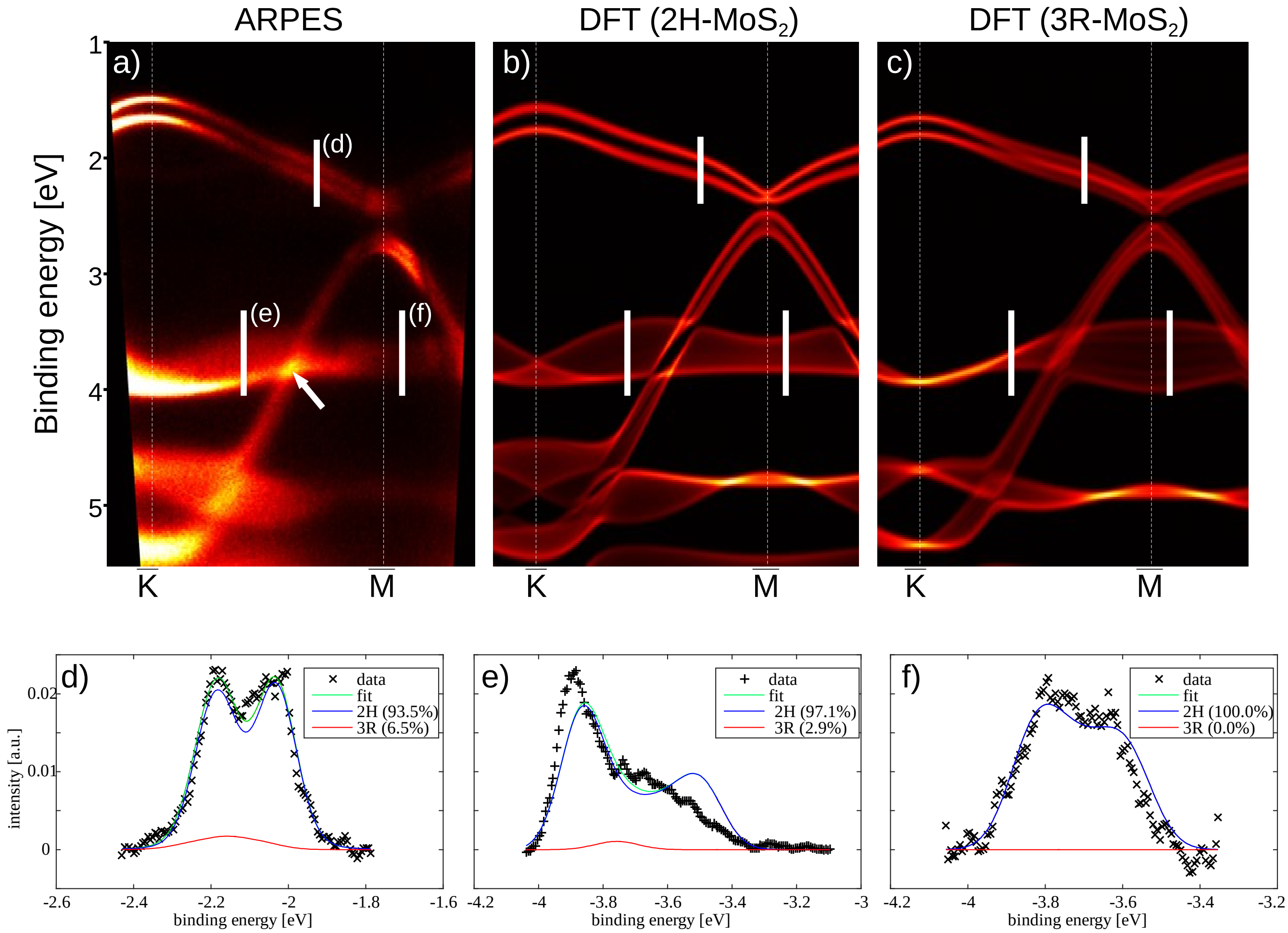

Figure 2. a) ARPES spectrum as it was shown in the main article, b) bulk-projected band structure of 2H-MoS_2 and c) 3R-MoS_2. White bars indicate the position of the EDCs d),e), and f).

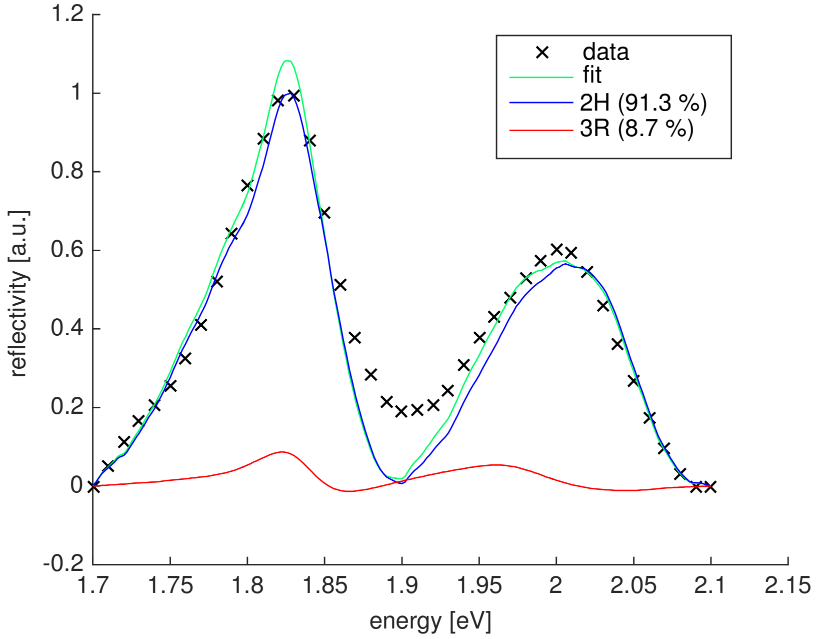

Figure 3. Reflectivity curve from our MoS_2 sample (x) and a fit (green) of a linear combination of two reference spectra from Ref.[1]

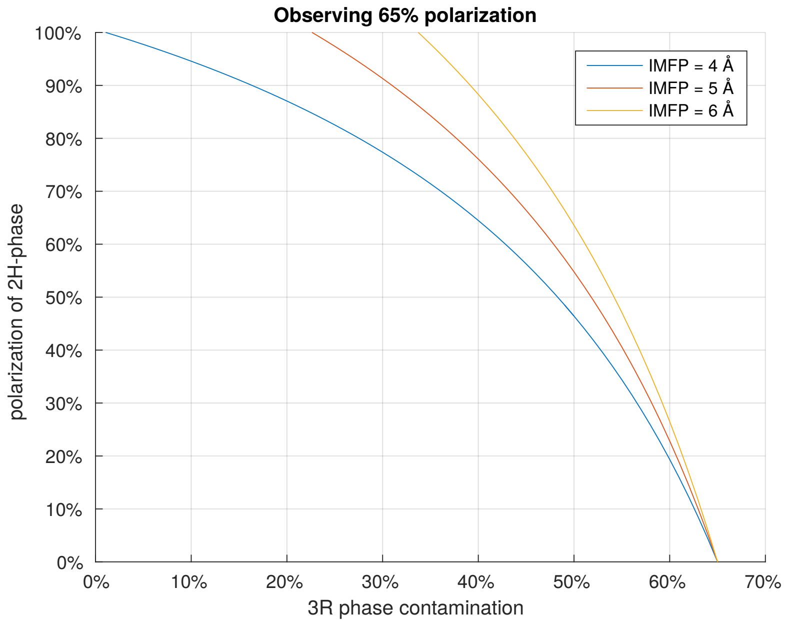

Figure 4. Spin-polarization in 2H-MoS_2 that can be concluded from observing 65% in ARPES as a function of the contamination of the sample by the 3R-MoS_2 phase for different IMFPs.
